# Nutrition in Pelvic Radiation Disease and Inflammatory Bowel Disease: Similarities and Differences

**DOI:** 10.1155/2014/716579

**Published:** 2014-05-28

**Authors:** Tiffany DeWitt, Refaat Hegazi

**Affiliations:** Research & Development Division, Abbott Nutrition, 3300 Stelzer Road, Columbus, OH 43219, USA

## Abstract

Due to the intestinal inflammation, tissue damage, and painful abdominal symptoms restricting dietary intake associated with both diseases, patients with intestinal pelvic radiation disease (PRD) or inflammatory bowel disease (IBD) are at increased risk to develop protein calorie malnutrition and micronutrient deficiencies. In the current paper, we review the nutritional management of both diseases, listing the similar approaches of nutritional management and the nutritional implications of intestinal dysfunction of both diseases. Malnutrition is prevalent in patients with either disease and nutritional risk screening and assessment of nutritional status are required for designing the proper nutritional intervention plan. This plan may include dietary management, oral nutritional supplementation, and enteral and/or parenteral nutrition. In addition to managing malnutrition, nutrients exert immune modulating effects during periods of intestinal inflammation and can play a role in mitigating the risks associated with the disease activity. Consistently, exclusive enteral feeding is recommended for inducing remission in pediatric patients with active Crohn's disease, with less clear guidelines on use in patients with ulcerative colitis. The field of immune modulating nutrition is an evolving science that takes into consideration the specific mechanism of action of nutrients, nutrient-nutrient interaction, and preexisting nutritional status of the patients.

## 1. Introduction

Inflammatory bowel disease (IBD) comprising Crohn's disease (CD) and ulcerative colitis (UC) is a major health problem. Approximately one million patients are diagnosed with IBD each year. IBD is thought to be more prevalent in industrialized parts of the world most notably in the United States and Europe [1]. However, recent analysis showed that both the incidence and prevalence of IBD are increasing with time and in different regions around the world, highlighting it as a global disease [2]. The exact etiology of IBD is unknown. However, it is thought to be multifactorial where genetics, diet, and environment play a role. IBD is a dysregulated immune response to the existing intestinal bacterial microbiota in genetically susceptible individuals. The exact pathogenesis of IBD is unclear but is thought to be a dysregulated intestinal mucosal response to intestinal luminal microbiota in genetically susceptible individuals. It is characterized by dysregulated T helper (Th) 1, Th2 cell immune responses, and other subsets of T cells, namely, Th17 and regulatory T cells [3]. Inflammation is different in CD from that of UC. Inflammation is confined to the colon in UC. In CD any part of the GI tract can be affected by inflammation. Patients with long-standing or refractory UC can undergo total proctocolectomy. Restorative surgery includes creation of an ileal pouch-anal anastomosis. Pouchitis, inflammation of the created ileal pouch, is the most common complication after restorative proctocolectomy. Pouchitis is characterized clinically by increased stool frequency, urgency, abdominal cramping, and discomfort.

Pelvic radiation disease (PRD) is transient or longer-term health problems, ranging from mild to very severe, that arise in noncancerous tissues as a result of radiation treatments to tumors of pelvic origin [4]. Noncancerous organ tissues that could be affected by radiation include the intestinal tract, urinary tract, bone, sex organs, or skin. Patients receiving radiation therapy for pelvic tumors frequently complain of GI-related symptoms that could be quality of life degrading. Symptoms of intestinal PRD include anal ulceration and bleeding, diarrhea, steatorrhea, hemorrhoids, nausea, and abdominal or anal pain [5]. Unfortunately, due to perception of embarrassment or thought that these symptoms are normal side effects of radiation treatment, patients may not inform their doctors about symptoms leaving most cases of intestinal PRD nondiagnosed and untreated. In this paper we will focus on the nutritional management of intestinal PRD resulting from the radiation associated injury to the GI tract. Similar to IBD, intestinal PRD is mediated by radiation-associated inflammation, oxidative stress, intestinal tissue damage, and edema.

## 2. IBD, Intestinal PRD, Diet, and Malnutrition

Malabsorption and maldigestion are common risks for developing malnutrition among patients with PRD and intestinal IBD due to the inherent damage of the GI tract. Left untreated, disease progression will further increase the nutritional risk as patients experience increasing symptoms such as pain, decreased dietary intake, and diarrhea. Extensive disease may require bowel resection as a necessary treatment which can further impair nutrient absorption and digestion. Changes in bowel function may impact the quality of life for PRD and IBD patients [6]. As a result, patients may decrease dietary intake or self-impose restricted diets in fear that eating will start or further aggravate symptoms. Patients benefit from nutritional counseling as they learn to manage varying phases of the diseases. Proper nutritional management can help manage nutritional risks.

Malnutrition has been well documented in IBD impacting 25–70% of patients and is more commonly observed in hospitalized patients [7]. The rate of malnutrition is less documented in PRD, but the documented weight loss can be an indicator of malnutrition, especially when persistent. The rate of weight loss in patients undergoing pelvic radiation can vary from 0 to 83% [8]. Prior to starting pelvic radiation, up to 32% of patients have lost >5% of body weight suggesting risk for malnutrition [8]. The many factors that contribute to malnutrition such as weight loss, decreased intake, absorption, or digestion and increased diarrhea, vomiting, or steatorrhea are present in patients with intestinal PRD leading to the assumption that malnutrition is present.

Malnutrition, or the risk of malnutrition, should be managed with proper screening, assessment, and nutritional interventions individualized to the patient's own nutritional and medical needs. The identification of nutritional risk can be completed by a validated screening tool such as the Nutrition Risk Screening-2002 (NRS-2002), Malnutrition Screening Tool (MST), or Malnutrition Universal Screening Tool (MUST) [9]. Certain screening tools have been validated in specific patient populations. For example, MUST is a validated tool to screen for malnutrition in the general population and in radiation oncology patients [10]. Nutrition screening tools are simple and quick questionnaires of the appetite changes and recent weight loss [9]. The goal of screening is to identify patients at risk of malnutrition who may benefit from further assessment and intervention [9]. If screening detects a risk of malnutrition, nutritional assessment should follow using tools such as the Subjective Global Assessment (SGA) or Mini-Nutrition Assessment (MNA) [9] to determine the best nutrition intervention.

GI inflammation is another common feature of both PRD and IBD. Intestinal biopsy samples of active IBD consistently reveal the evidence of inflammatory cell infiltrates and other markers of inflammation [11]. In patients receiving radiation to the pelvis, inflammation of the small bowel may only develop in more than 70% of patients [12]. Inflammation creates a catabolic state requiring an increased dietary intake to meet the increased energy demands. Intestinal inflammation further contributes to malnutrition risk by exacerbating symptoms such as rectal bleeding, severe diarrhea, abdominal pain, fever, and weight loss [11].

A low residue diet is thought to help reduce fecal volume and is characterized by moderating fiber, milk, and meat products [13]. However, low residue diets can be nutritionally inadequate and are not recommended for long periods of time [13]. If a low residue diet is recommended, enteral formulas that provide elemental and complete balanced nutrition are preferred [13]. Low residue diets pose a risk with the potential lack of the beneficial effects of dietary fiber. The intake of dietary fiber can help alleviate diarrhea by increasing fecal mass and modulating GI motility [14]. Insoluble fiber acts as a stool bulking agent due to the inability to be digested whereas soluble fiber plays a role in improving the integrity of gut microbiota [14]. Fiber plays an important role in fecal formation and can also play an active role in maintaining healthy microbiota environment in the terminal ileum and colon. Collectively, dietary fiber can be beneficial to patients with intestinal PRD and IBD in many ways and does not need to be completely eliminated.

Low fat diets, defined as fat providing 25% of total daily calories, are generally recommended to alleviate diarrhea in both PRD and IBD [8, 15, 16]. Consistently, a recent Cochrane review of the literature has shown that dietary modifications to the type of fat, restrictions to fat and lactose, and supplementation of fiber during pelvic radiotherapy can help decrease the amount of diarrhea [17]. While minimizing fat intake, the type of fat can also help in the management of inflammation, a topic that will be discussed later in this paper. Different types of fats have also been shown to be either beneficial or detrimental to bowel health due to pro- or anti-inflammatory properties and the ease of digestion and absorption. For example, medium chain triglycerides do not require bile, chylomicrons, or carnitine for their absorption and are therefore a more readily available source of energy as compared to long chain triglycerides.

Micronutrients play an important role in meeting the nutritional needs, maintaining homeostatic immune, and providing antioxidant functions. While researchers note varying degrees of vitamin and mineral deficiencies in both diseases, there are no consensus recommendations regarding routine supplementation of vitamins and minerals [8, 15, 18]. Specific vitamin and mineral deficiencies vary depending on the acuity of the diseases. Fat soluble vitamin absorption may be impaired when steatorrhea is present. Vitamin D deficiency has been found in IBD patients and is suggested to contribute to the risk of developing disease-associated osteopenia [19]. Antioxidant vitamins C and E have been discovered to have additional needs during times of inflammation in CD patients [20, 21]. In a randomized controlled trial, supplementation of vitamin E at 800 IU and vitamin C at 1000 mg was shown to be effective in increasing serum levels of Vitamins C and E while helping to reduce oxidative stress over a 4-week period [21]. The significant reduction in oxidative stress was found by measurements of breath pentane and ethane, plasma lipid peroxides, and F2-isoprostane [21]. Supplementation of vitamins C and E has also shown favorable outcomes in patients with chronic radiation proctitis in a small, nonblinded study [22]. In the study, patients consuming 400 IU of vitamin E and 500 mg of vitamin C orally three times per day for 4 weeks reported alleviation of some symptoms including diarrhea (*P* < 0.001) [22]. The study also highlighted the significant prevalence of GI symptoms that impacted 80% of the study participants with chronic radiation proctitis [22]. When deficiency is expected, vitamin and mineral supplementation may be considered to replenish and maintain optimal levels.

When making dietary modifications, it is important to minimize any food restrictions in order to ensure enough protein and calories are being consumed to prevent weight loss and malnutrition. These goals can be achieved through diet and with the help of oral nutritional supplementation (ONS), enteral tube feeding, or a combination of all. For instance, low fat, low residue, high protein, and calories diets are recommended for patients with PRD and IBD during the active inflammatory phases [8, 16]. Protein recommendations of patients in the quiescent noninflamed state are 0.8 g/kg but can increase to 1.0–1.5 g/kg during active inflammation [23]. It may be difficult for patients to achieve this high intake level through diet alone. Consistently, ONS can be useful in helping patients consume enough calories and protein to meet their elevated nutritional needs given the catabolic state of inflammation induced by radiation or active disease. ONS should be considered when patients are at risk for malnutrition [24].

## 3. Role of Nutritional Therapy in the Management of Intestinal PRD and IBD

### 3.1. Nutritional Modulation of the Intestinal Microbiota

Another common feature of both intestinal PRD and IBD is the associated dysregulation of gut microbiota (dysbiosis) [25, 26]. Dysbiosis is known to be present in IBD, both CD and UC, and contribute to the associated dysregulated immune response [25]. IBD patients with active disease show altered microbial diversity [27] with an increase in pathogenic microbes such as* Escherichia coli* as compared to healthy control subjects [28]. It is suggested that the intestinal dysbiosis may also be present in intestinal PRD [26]. When the gut incurs injury or insult, the gut microbiota environment can be altered leading to decreased protection by the beneficial strains and an expanded pathogen population leading to increased risk of infection. Consistently, preclinical studies have suggested that intestinal bacteria have the ability to influence the intestinal response to radiation injury [14]. 

According to the WHO definition, probiotics are live microorganisms which, when administered in adequate amounts, confer a health benefit on the host, although this definition is debated by other institutions. Prepopulating or repopulating the gut microbiota with probiotics to prevent or repair intestinal damage is of particular therapeutic interest in intestinal PRD and IBD. It is important to understand that the probiotics category includes a plethora of microbial entities that differ in their strain, strength, and mechanism of action. For example, some probiotics contain a single strain of bacteria; others contain a mix of different bacterial strains while other probiotics are yeasts in nature. Within each of these probiotics strains, different strengths exist. Given the heterogeneity of these beneficial microbes, it is difficult to show consistent clinical effects of probiotics as a group in different disease settings. Another determining factor for the effectiveness of probiotics is the unknown diversity of the patient's own intestinal microbiota. Research will continuously evolve to determine the most therapeutic uses of probiotics depending on the patient's own microbiota distribution. For the aforementioned reasons, a review of the literature by Fedorak and Demeria showed mixed results in the efficacy of probiotic therapy inducing and maintaining remission in various IBD conditions [25]. Meanwhile, research of the effectiveness of specific probiotics in a specific disease setting could be more consistent. For instance, the use of the probiotics strain mix VSL#3 for preventing and maintaining remission of pouchitis, and in the maintenance of remission in UC, has been more consistently appreciated and a consensus for “Probiotic Use—2011 update” recommended their use for these specific indications [29]. Similarly, results demonstrating the beneficial effects of probiotics for the prevention and treatment of radiation induced diarrhea have been shown in a recent meta-analysis [30]. Given the fact that probiotics should be continuously enterally administered to colonize the gut and exert their effects, the long term benefit of the probiotics has yet to be proven.

Prebiotics are nondigestible carbohydrates that could enhance the proliferation and/or metabolic activities of the beneficial intestinal microbiota. Examples of prebiotics are soluble fibers and the inulin-like fructans such as fructooligosaccharides (FOS) and galactooligosaccharides (GOS) [14]. One of the mechanisms of the action of prebiotics is the release of short-chain fatty acids (SCFAs) upon fermentation by the intestinal microbes. SCFAs not only are essential for the integrity of the colonic epithelial cells but also exert anti-inflammatory effects in models of IBD [31]. They also create an acidic intestinal luminal environment that is unfavorable to pathogenic bacteria [14].

Collectively, one of the major goals for nutritional management of patients with PRD and IBD should be correcting the imbalance in the gut bacterial environment to help reduce inflammation and stimulate healing either by the use of probiotics or prebiotics or their combination, synbiotics [8, 32].

### 3.2. Nutritional Modulation of Inflammation

The marine omega-3 fatty acids have proven to modulate several aspects of inflammation in preclinical models [33, 34]. Furthermore, the long chain omega-3 fatty acids eicosapentaenoic acid (EPA) and docosahexaenoic acid (DHA) have been shown to exert anti-inflammatory effects in certain diseases [35, 36]. Their mechanism of action appears to occur at the membrane level, suggesting that proper manipulation of the immune cell membrane fatty acid composition can potentially have a profound influence on the health of the individual [36]. The increased consumption of the EPA and DHA from fish oil results in an increased proportion of these fatty acids in the cell membrane, displacing arachidonic acid (AA), a precursor of the more proinflammatory eicosanoids [37]. The impact of the immune modulating effects of EPA and DHA goes beyond its effects on the structure of the cell membrane and includes decreased production of inflammatory components by altering gene expression through direct actions on intracellular signaling pathways [37]. An example of this benefit has been documented in studies of fish oil-supplemented oral nutrition supplements in patients with IBD [38]. Wiese et al. studied the ability of supplementing two 8-ounce cans of an ONS containing fish oil, antioxidants, and prebiotics in CD patients to modulate disease severity [38]. One finding of the study was a significant reduction in Crohn's Disease Activity Index CDAI in patients with the higher EPA levels (*P* = 0.005) [38]. The study showed that specially designed nutrition formulas with fish oils and elevated antioxidants may offer anti-inflammatory benefits over standard formulas [38]. Importantly, the results cannot be attributed to one unique ingredient but highlight the synergistic benefits of supplementing ONS with immune modulating nutrients like fish oil. The clinical benefit of fish oil supplementation per se and the effects on disease relapse have been studied in randomized control trials in IBD patients and have shown inconsistent results [39, 40].

### 3.3. Nutrition as Therapy

Given its immune modulating and inflammation attenuating effects, enteral nutrition is used not only to treat malnutrition and support the nutritional status but also to alleviate the burden of the inflammatory disease in patients with intestinal PRD and IBD. Exclusive enteral nutrition has been clinically shown to induce remission and mucosal healing in pediatric CD, but not UC, [41, 42]. The therapeutic goal of exclusive enteral feeding is to promote mucosal healing generally assessed by the alleviation of symptoms. The mechanism of action of enteral nutrition inducing remission is still unknown; however, it could be hypothesized that enteral nutrition provides a combination of nutrients that help restore the intestinal microbiota environment, reduce inflammation, and provide nutrients necessary for proper cell signaling [43]. Compliance to exclusive enteral nutrition is the key to increasing the likelihood of symptom relief and remission. Whether oral or tube fed, exclusive enteral nutrition has been clinically shown to induce readmission in patients with CD [16, 44–48]. Clinical recommendations from the North American Society for Pediatric Gastroenterology, Hepatology, and Nutrition (NASPGHAN) support the use of exclusive enteral nutrition as primary therapy for the treatment of pediatric patients with CD [49]. These positive results in CD have lead researchers to consider enteral nutrition for PRD patients, but clinical studies are warranted.

Elemental diets have been shown to help maintain IBD remission by replacing half of daily caloric intake for at least one year [16, 41, 50]. Consistently, elemental diets have shown positive trends in helping patients with PRD and IBD in the management of the disease [51]. An elemental diet may contain partially hydrolyzed proteins along with other easily absorbed nutrients including medium chain triglycerides and prebiotics. In patients with PRD (radiation enteritis), elemental diets were shown to help alleviate diarrhea and potentially help decrease risk of chronic enteritis [51]. In IBD, elemental diets have been shown to be beneficial in inducing remission; however, a meta-analysis comparing the efficacy of elemental versus polymeric formulas failed to show differentiation [32, 43]. The use of elemental formulas is a clinical decision that is based on the patient's GI absorptive function and tolerance to polymeric formulas.

Enteral feeding maintains the trophic, immune, and hormonal responses of the intestinal epithelium and stabilizes the gut barrier function thereby decreasing the rate of complications associated with parenteral feeding, especially infections. Every attempt should be exerted to manage the EN-associated GI intolerance including slowing of the rate of feeding, decreasing the volume fed, and increasing the duration before advancing to goal and switching to elemental diet feeding before parenteral nutrition. When enteral-feeding associated intolerance becomes severe enough to justify cessation of enteral feeding, parenteral nutrition is warranted to avoid malnutrition and its associated adverse effects on clinical outcomes [16, 43]. Other indications of parenteral nutrition in patients with IBD include the short bowel syndrome as a result of extensive intestinal resection.

## 4. Conclusion

Intestinal PRD and IBD share common pathologies including intestinal inflammation, tissue damage, and intestinal microbial dysbiosis. Malnutrition is common in both diseases and should be properly diagnosed and managed. Nutrition plays a major role not only meeting the nutritional needs for patients with both diseases but enteral nutrition exerts immune modulating effects that could help optimize the management of the two diseases. Exclusive enteral nutrition has been recommended for induction of remission of pediatric CD. The literature of immune modulating nutrition in the disease management of IBD and intestinal PRD is still an evolving topic that should take into consideration not only the type and dose of the nutrient but also the preexisting nutritional status and nutrient-nutrient interaction. The current literature supports the beneficial role of specific multistrain probiotics in patients with pouchitis and in the maintenance of remission of ulcerative colitis while the immune modulating effect of fish oil-supplemented enteral nutrition is an interesting topic for further research.
